# The Somatic Rubber Hand Illusion does not modulate perceived hand position in children with severe visual impairments

**DOI:** 10.1038/s41598-026-48413-6

**Published:** 2026-04-26

**Authors:** Carolina Tammurello, Lara Audrey Coelho, MariaBianca Amadeo, Walter Setti, Claudio Campus, Sabrina Signorini, Federica Morelli, Elena Cocchi, Sandra Strazzer, Francesca Tinelli, Paola Camicione, Massimiliano Serafino, Monica Gori

**Affiliations:** 1 Unit for Visually Impaired People (U-VIP), Italian Institute of Technology, Genoa, Italy; 2 Department of Informatics, Bioengineering, Robotics and Systems Engineering (DIBRIS), University of Genoa, Genoa, Italy; 3 Developmental Neurophthalmology Unit, IRCCS Mondino Foundation, Pavia, 27100 Italy; 4 David Chiossone Foundation, Genoa, Italy; 5 Scientific Institute IRCCS Eugenio Medea, Bosisio Parini, Italy; 6 Department of Developmental Neuroscience, IRCCS Fondazione Stella Maris, Pisa, Italy; 7 Division of Ophthalmology, IRCCS Istituto Giannina Gaslini, Genoa, Italy; 8 Autism Spectrum Disorders Service, Department of Psychiatry, Lausanne University Hospital and University of Lausanne, Lausanne, Switzerland

**Keywords:** Neuroscience, Psychology, Psychology

## Abstract

In the Somatic Rubber Hand Illusion (SRHI), synchronous brushing of a participant’s hidden hand and a dummy hand induces proprioceptive drift, a shift in perceived position of the hand towards the dummy hand, along with the sensation of brushing one’s own hand. While sighted individuals experience proprioceptive drift since childhood, blind adults are immune. Here, we tested Severely Visually Impaired (SVI, n = 13) children and sighted controls (n = 44) with the SRHI. Surprisingly, both groups subjectively reported illusory self-touch. We posit that children with severe visual impairment tentatively infer a common cause when experiencing synchronous stroking of both a dummy hand and their own hand – just like sighted children do. However, unlike sighted children, their perceived hand position is not updated accordingly. Contrary to our hypothesis, we found that only in the SVI group proprioceptive precision predicts drift values, as well as illusory self-touch, with higher precision leading to larger drifts and a stronger illusion. We suggest that higher proprioceptive competence is required for SVI children to develop sighted-like responses to the illusion and overcome difficulty in re-mapping body position onto external space based on multisensory stimulation.

## Introduction

Developmental vision plays a pivotal role in shaping multisensory integration^1–6^ and has been proposed to calibrate other sensory modalities in tasks such as haptic orientation discrimination^7^ or auditory spatial bisection^8^. In the absence of visual experience, perceptual development follows a distinct trajectory. Proprioception, i.e., the ability to sense the position, movement, and spatial orientation of one’s own body parts, is thought to rely on visual calibration during development. Accordingly, proprioceptive impairments have been reported in blind children and adults^9^. However, findings are not uniform: enhanced proprioceptive acuity at the ankle joint has also been observed in blind individuals, although this improvement did not translate into superior postural control^10^, and blind adults and children exhibited improved size discrimination precision in the audio–haptic condition when auditory cues were congruent with object size^11^. Taken together, these mixed findings suggest that the development of proprioception in the absence of vision may involve both compensatory and maladaptive mechanisms. Critically, however, it remains unclear how own body localization in space is modulated by other sensory modalities in children with Severe Visual Impairments (SVI). Multisensory modulation of perceived hand position in space is strikingly demonstrated in the sighted population, but not in blind adults, by the Rubber Hand Illusion (RHI). During RHI, synchronous tactile stimulation is applied to a visible dummy hand and one of the participant’s hidden hands, and the perceived location of the participant’s stimulated hand shifts towards the dummy hand —an effect referred to as proprioceptive drift^12–14^; in other words, synchronous visuo-tactile stimulation induces a recalibration of perceived hand position. Additionally, participants report to perceive ownership over the dummy hand and referral of touch, a feeling correlated with that of ownership^15^ whereby tactile sensations felt on their real hand are mislocalized to the dummy hand. In the somatic version of the RHI (SRHI), which does not require vision, the experimenter guides a blindfolded participant in brushing a dummy hand, while synchronously delivering an identical stimulation to the participant’s other hand^16^. This creates a conflict between haptic and proprioceptive information. Haptics conveys the spatial-temporal coherence of the touches delivered to real and dummy hands, thus favoring the merging of the two hands in a unitary percept^17–19^; that is to say, if the paradigm is successful, the participant would erroneously perceive to be touching their own hand^16^. We will refer to this subjective experience induced by SRHI as illusory self-touch^20,21^. Instead, proprioception signals the spatial discrepancy (i.e., the distance between the hand delivering the stimulation and the one receiving it), thus counteracting the illusory self-touch. Accordingly, larger spatial discrepancies counteract the illusion in both the visual^22–24^) and the SRHI paradigm^25^. Nevertheless, at approximately 15 cm, the similarities between the stimuli delivered to the dummy hand and the real one constitute a sufficient amount of evidence for the brain to ‘merge’ -albeit partially- their locations^26,27^, provided that certain spatial-temporal constraints (i.e., synchrony and limited inter-manual distance) are met^28,29^. In line with the interpretation of a merging of the two hands, SRHI, like RHI, induces proprioceptive drift. It is worth noting that proprioceptive drift in SRHI can also be aided by auditory information: Radziun and Ehrsson found that proprioceptive drift, as well as subjective ownership and illusory self-touch, are significantly increased by the addition of synchronous auditory cues in a SRHI paradigm^30^, suggesting that synchronous sounds are taken as further evidence that the two hands are one and the same. Consistent with the idea that auditory sensory evidence can shape body-representation, studies in both adults and children show that action-related auditory feedback can recalibrate proprioception and alter perceived body structure^31,32^. Both proprioceptive drift and referral of touch following RHI have been reported in children as young as four years old^33–36^. In the only SRHI study conducted with children, Nava et al. (2017) demonstrated that the somatic paradigm is likewise effective in children, eliciting illusory self-touch as well as proprioceptive drift^37^. Notably, Nava showed that proprioceptive recalibration (i.e., proprioceptive drift) was larger in children aged 8–9 y.o. than in the 4–5 y.o. group, in a reversal of the pattern observed with the visual paradigm, where a stronger visual capture of proprioception in childhood gradually decreases toward adult levels by around 10 years of age^35,38^. These differential weighing of different senses during development^35,37^, taken together with the adherence to the spatial rule and the temporal rule already mentioned^22–24^, all point to RHI and SRHI being the product of multisensory integration mechanisms^39^. In accordance with this, subjective ownership induced by RHI has been conclusively linked to sensory signals, with proprioceptive variability systematically shaping ownership judgments in line with signal-detection-theory principles^40^. Also in line with multisensory integration frameworks, experimentally increasing proprioceptive noise via tendon vibrations have been shown to increase the subjective experience of ownership^41^. Despite this, it remains unclear whether proprioceptive drift (or its absence) is connected to individual levels of proprioceptive precision - i.e., the consistency of participants’ responses across repeated trials - when localizing their own unseen hand^42^. The present study aims to test how the SRHI induces proprioceptive drift and illusory self-touch during development and how they relate to individual proprioceptive precision within the framework of multisensory integration. To this end, we performed our first experiment in sighted school - children. Based on^37^, we predicted that proprioceptive drift would increase with age. We then hypothesized that if proprioceptive drift is driven by bottom-up multisensory integration mechanisms, individual levels of proprioceptive precision would predict the extent of proprioceptive drift in different participants, meaning that highly precise children would rely more heavily on their proprioception and therefore be less prone to re-calibration. Additionally, we included a condition with auditory cues, to test whether auditory information contributes to recalibration in this age group, and if the effect begins at a specific age^43,44^. While the effect of SRHI has been documented in the sighted population across the lifespan, SRHI has consistently failed to elicit illusory self-touch in blind adults, or to induce proprioceptive drift^5,20,45^.However, the mechanisms underpinning such ‘immunity’ remain unclear. Conceivably, their proprioceptive stability may stem from a finer tuning of proprioception during development compared to sighted individuals, owing to cross-modal plasticity. In fact, this population has been shown to develop a heightened precision in the spared senses^2,46^; in the context of the SRHI, a higher proprioceptive precision may simply render them too sensitive to the spatial discrepancy between the two hands to be deceived. Accordingly, this population was shown to be immune to illusory phenomena such as the ventriloquist effect^47^ and SRHI^5,20,45^,which indeed arise from erroneous merging of separate stimuli and may reasonably be impeded by a lower tolerance to spatial-temporal discrepancies. If this was the case, we may observe a different pattern during childhood, as the rapidly developing bodies of children may lead to a noisier estimate of hand position^48,49^. Experiment two aimed to test the response of Severely Visually Impaired (SVI) children to the SRHI. Considering a possible null finding, we devised a paradigm that delivered complex sensory stimulation, both through variable movement patterns and duration^50^, and through the presence of sounds in the condition with auditory cues. We speculated that if the effect of illusory self-touch and proprioceptive drift were absent in the tactile only condition, the added complexity in the sensory stimulation provided by a spatially and temporally coherent sound may constitute sufficient evidence for them to attribute the stimuli to a common cause^28^, thus inducing proprioceptive drift and illusory self-touch. Finally, to maximize the effectiveness of the paradigm, the children were purposefully kept unaware of the experimental setup, so as to prevent them from attempting self-corrections when performing hand localizations.

## Methods

The experimental procedures reported below were identical to those previously validated for eliciting the proprioceptive drift and illusory self-touch in^51^. The current study extends this work by examining the contribution of visual experience and the factors driving the illusion.

### Participants

Sighted and Severely Visually Impaired children aged between 6 and 11 took part in the study. The research protocol was approved by the ethics committee of the local health service (Comitato Etico Regionale, ASL3 Genovese, Italy) and conducted in line with the Declaration of Helsinki. Written informed consent was obtained from a parent and/or legal guardian prior to testing, and verbal assent was obtained from the participants themselves.

#### Sighted children

The control group was recruited from a local school^51^. Children with a known diagnosis of autism and/or intellectual disability were excluded from the study. A total of 52 sighted children aged 6 to 11 years old took part in the study, only one had to be excluded as non-compliant; thus, the control group was composed of 51 children. This age range was chosen as its middle-point, the age of 8 has been shown to be a landmark for development of multisensory integration^43^. Visual inspection of the data led to the further exclusion of seven participants from the dataset as they were an outlier in at least one experimental condition; further checks ascertained that all excluded participants displayed proprioceptive drift values that fell +−2 SD away from the mean of their age group in at least one condition. 44 sighted participants were thus included in the final analyses.

#### Severely visually impaired children (SVI)

All the SVI children who took part in this study had congenital visual impairment. The children were recruited at the Developmental Neuro-ophthalmology Unit of IRCCS Mondino Foundation, Pavia, Italy, and were tested on the premises (for an overview of causes of visual impairment and visual acuity, see Table 1). A total of 16 SVI children aged 6 to 11 years old took part in the present study; however, three were excluded from the analyses: two due to non-compliance with the instructions, and one because they had difficulty understanding the instructions. 13 SVI children were therefore included in the analyses (reported in Table 2); 10 of these children completed all trials. SVI children were recruited if their visual impairment was due to affections of the peripheral visual system. We recognize that this is a small sample to draw conclusions from and therefore present our findings as preliminary. Children were excluded in case of cerebral involvement or developmental delay/intellectual disability. In accordance with Italian law, children were categorized as low vision if their visual acuity measured at 3 meters was between 3/10 and 0.05/10; blindness was defined as a best-corrected visual acuity worse than 1.3 LogMAR, according to the World Health Organization’s criteria and Italian law(G.U. Serie Generale, n. 93 del 21 Aprile 2001). An overview of age and sex distribution across groups is displayed in Table 1.

**Table 1:** Age, cause of visual impairment, category and residual vision (if applicable) of SVI children included in the analyses. Visual acuity is reported both in decimal and logMAR scale.

Part-icipant	Age (years)	Cause of visual impairment	Category	Decimal Visual Acuity (3m distance)	LogMAR Visual Acuity (3m distance)
S01	8.05	Inherited retinal dystrophy	Blind	No answers	No answers
S02	5.91	Inherited retinal dystrophy	Blind	No answers	No answers
S03	9.49	Inherited retinal dystrophy	Low vision	2/10	0.7
S04	8.49	Inherited retinal dystrophy	Low vision	0.5/10	1.3
S05	7.67	Congenital cataract	Low vision	1.5/10	0.8
S06	9.60	Inherited retinal dystrophy	Blind	No answers	No answers
S07	6.94	Congenital glaucoma	Blind	No answers	No answers
S08	7.48	Inherited retinal dystrophy	Blind	No answers	No answers
S09	11.03	Anophthalmia	Blind	No answers	No answers
S10	6.58	Inherited retinal dystrophy	Blind	No answers	No answers
S11	6.41	Retinopathy of prematurity	Blind	No answers	No answers
S12	6.48	Inherited retinal dystrophy	Blind	No answers	No answers
S13	10.36	Inherited retinal dystrophy	Blind	No answers	No answers

**Table 2:** Overview of the participants included in the analyses divided in age groups.

Age group	6 y.o.	7 y.o.	8y.o.	9 y.o.	>10 y.o.	Total
Sighted children	11	8	9	7	9	44
SVI children	5	2	2	2	2	13

### Materials

**Fig. 1:**
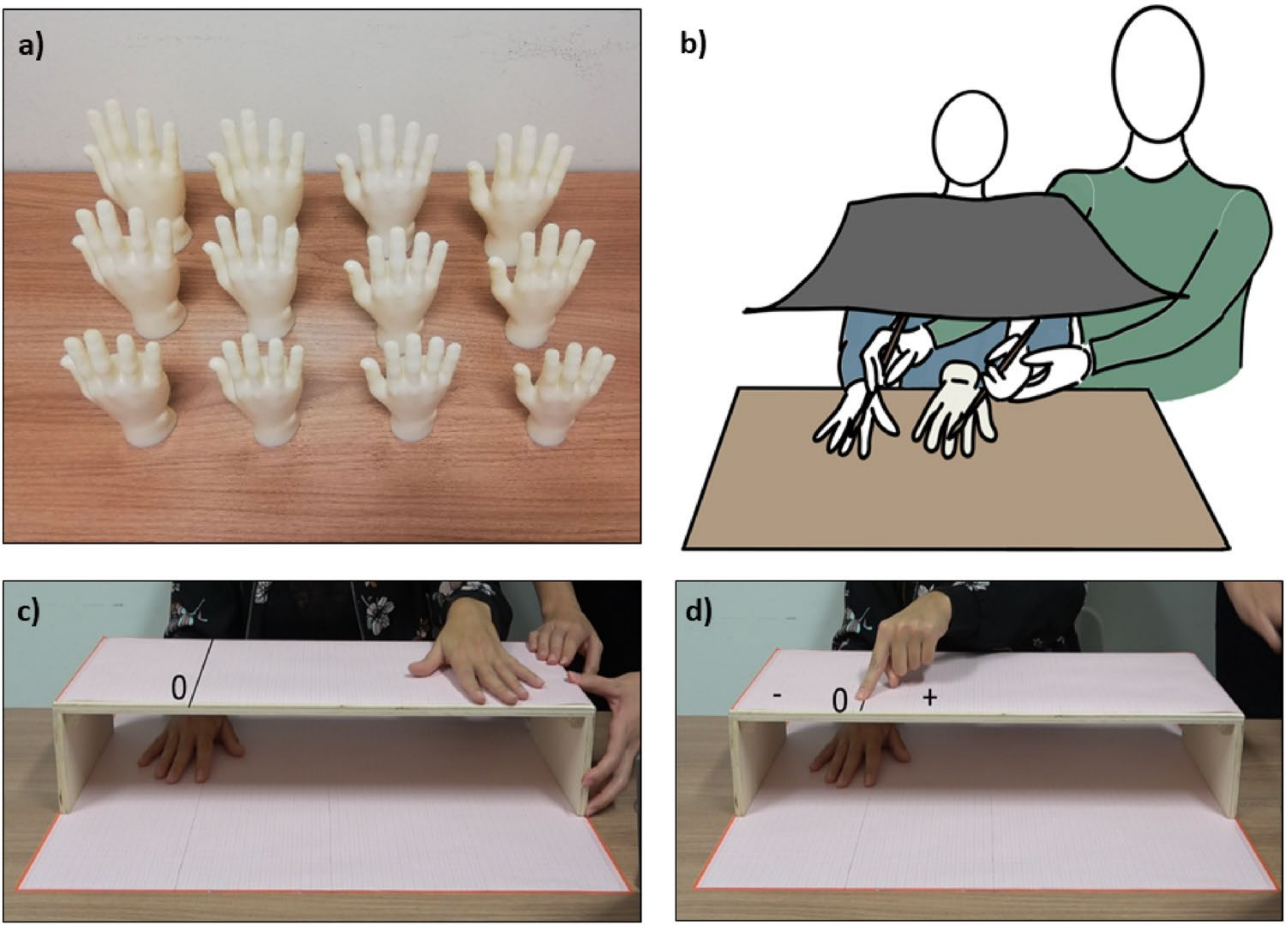
Materials used to carry out SRHI. Figure a: some of the dummy hands used. Figure b: a pictorial representation of the bib showing how participants’ sight of their hands was covered during illusion induction. The hem of the bib was taped to the wall in front of the participant. Figure c: the participant’s hand is placed on the small table prior to the pointing task. Figure d: the pointing task is performed. Perceived position was recorded as the distance from pointed location and the black line - signaling the correct finger location. As such, perceived position was 0 if the location pointed at was above target; positive if the location fell to the target’s left (towards the center of the table); and negative if it fell to the target’s right.

To carry out the Somatic Rubber Hand Illusion task, a set of 30 3D-printed plastic hands, depicted in Fig. 1a, was used. The dummy hands ranged from the size of a one-year-old child to that of a grown man; for a full description, see^52^. During a single experimental session, the dummy hand whose size best matched the size of the participant’s hand was used. A custom-made wooden table of 21x59,5 cm and 10 cm tall was placed on a desk and used to perform the pointing task. A gridded A2 paper sheet (with same width as the wooden table) taped to the desk was used to mark the exact position of the participant’s right index finger, while another gridded sheet was taped to the wooden table to mark the perceived position of said finger during the pointing task.

During the illusion induction phase, a set of earphones and two identical round size 10 paint brushes were used. The choice to deliver tactile stimulation through brushes as opposed to gloved hands was made after careful consideration for the following reasons: 1) because holding a brush would make it less likely for children to accidentally hit the dummy hand (making a noise incompatible with that of a human hand), thus interfering with the illusion; 2) because having to wear rubber gloves was expected to reduce task compliance; and 3) holding the brush reduced the likelihood that SVI would tactilely explore the setup if they experienced a discrepancy in perceived hand position induced by the task.

Finally, a black shaving bib with padded shoulders (depicted in Fig. 1b) was used to cover the sight of the participant’s hands. This solution was preferred to a blindfold as it ensured the participant did not receive any visual feedback and was deemed more respectful of low vision children.

### Procedure

Each participant sat at a desk where the small wooden table was placed in front of them. Sighted controls and low vision children were made to wear the bib, whose hem was attached to the wall in front, so as to cover the sight of the participants hands (see Fig. 1b). To record the participants’ perceived position of their right index finger prior to SRHI, three pointing trials (i.e. the baseline condition) were first performed. After recording the baseline, each participant performed two experimental conditions: tactile SRHI and audio-tactile SRHI, which were carried out sequentially and counterbalanced across participants. Three illusion induction trials were performed per each condition. Following each illusion induction trial, one pointing trial and one survey (see Table 3) were performed, for a total of nine pointing trials per participant (three baseline pointing trials, three pointing after tactile SRHI, three pointing after audio-tactile SRHI) and six surveys. At the end of the entire experiment, the participant was asked a final question to measure their suggestibility (see Table 4). Between each condition and the next, the participant was invited to take a break and move their arms and hands, so as to prevent habituation of their position sense. It should be noted that the primary focus of the present study was on proprioceptive drift. For this reason, we chose not to inform our participants of the experimental set-up until after the experiment was concluded. In fact, prior knowledge of the RHI paradigm could arguably induce individuals to attempt to self-correct when pointing to their hand’s perceived location (a measure from which we would then derive proprioceptive drift), as participants may guess that the paradigm is designed to induce a specific error (to point towards the dummy hand instead of the real one). It was therefore crucial for us to ensure that our participants – SVI ones in particular – would not enact any such strategy.

#### Pointing task

**Fig. 2:**
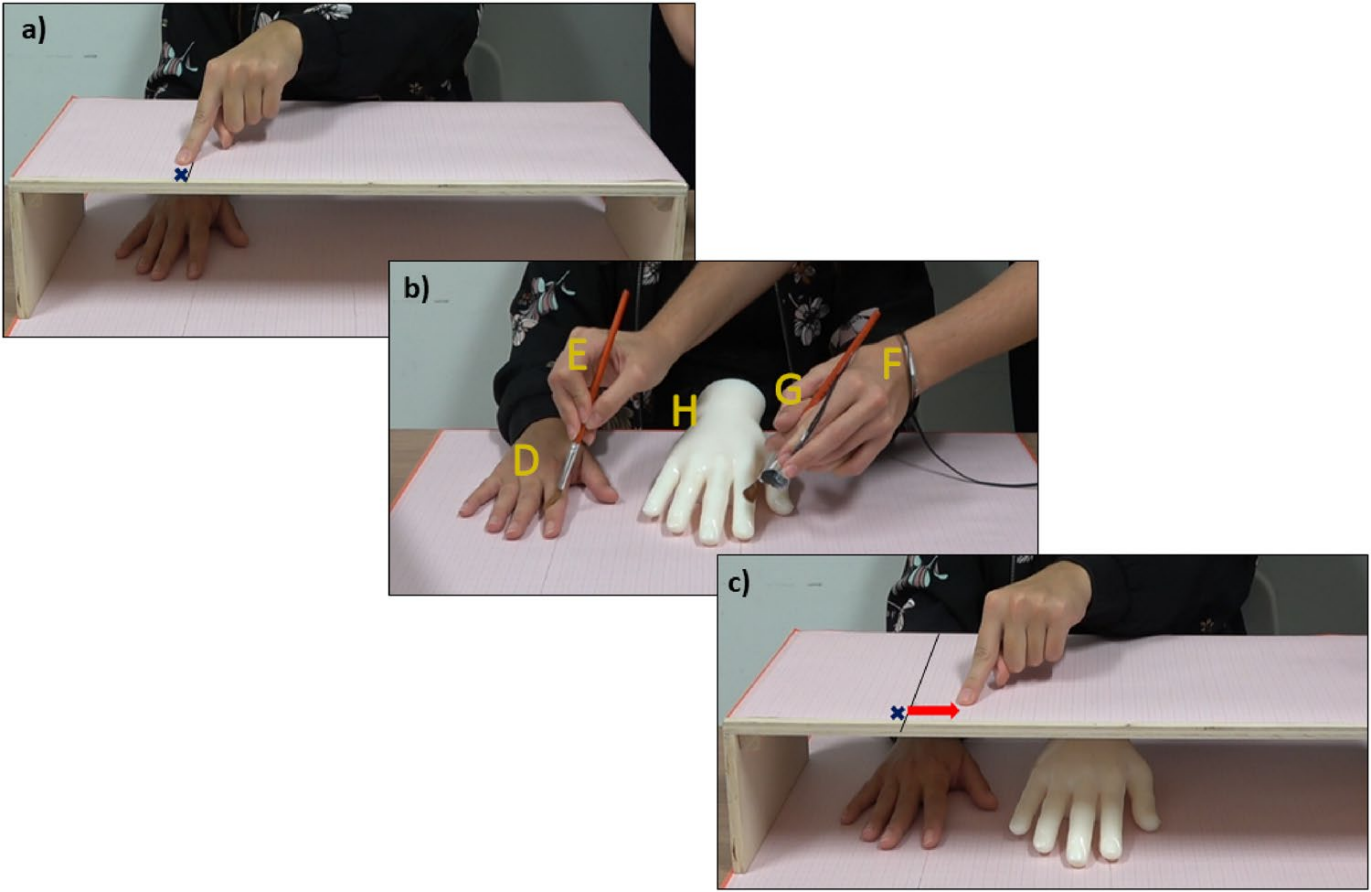
Proprioceptive Drift induced by SRHI. a: one o)f three pointing trials performed to record the participant’s baseline perception. Figure b): a session of audio-tactile SRHI is performed, with the participant’s right hand (D) brushed by the experimenter’s right hand (E) while the left hand of the experimenter (F) guides the participant’s guide the participant’s left hand (G) in brushing the dummy hand (H). Figure c): one of three pointing trial performed after audio-tactile SRHI (one trial after each SRHI session); the red arrow highlights the shift between perceived position before and after SRHI.

Each participant sat at a desk and wore the bib. The small wooden table would then be placed above their hands, with its center aligned to the body midline. After exploring the wooden table with both hands, the participant’s right hand was placed on the desk so that the index finger would fall fifteen cm to the right of their body midline; importantly, the participant was never allowed to see where their hand had been positioned. Their left hand was then placed atop the wooden table in a comfortable position (Fig. 1 c), which would be marked as the starting point for all their pointing trials. The participant was then asked to use their left hand to point at the spot on the table under which they perceived their right index finger was placed (Fig. 2). This pointing procedure was repeated three times to capture the subject’s baseline and one time after each illusion induction trial.

#### Induction of rubber hand illusion

Tactile stimulation was delivered via brushstrokes of two possible durations (300 and 500 ms approximately) in randomized order and two possible directions (upwards and downwards along the pointer) within each trial. In the audio-tactile condition each tactile stimulation was paired with an auditory cue which was synchronous and spatially congruent, i.e., the sound source was placed near the point of contact with the dummy hand and moved along with the brush employed for the stimulation.

The selected dummy hand was surreptitiously positioned at the center of the table, so that its index finger would be aligned to the participant’s body midline. The participant was then asked to hold the paintbrush with their left hand and to let the experimenter guide them. The experimenter made the participant brush the dummy hand’s index finger, while synchronously brushing the participant’s own right index finger with the other brush. Each trial lasted 42 seconds, with brushing frequency = 1 Hz and two possible brushing durations of around 300 ms or 500 ms. The induction duration of 42 seconds was considered sufficient to elicit both illusion and proprioceptive drift^36^ without resulting in a lengthy experimental paradigm. In order to follow the described rhythm and durations, the experimenter followed Matlab-generated visual cues on the computer screen. The brushing on the real and dummy hand was delivered synchronously and consistently in both direction (which could be either towards or away from the tip of the finger) and duration (300 or 500 ms).

The audio-tactile SRHI was done the same way as the tactile condition, with an added acoustic stimulation. To deliver said stimulation in a space-relevant manner, an earplug was taped to the experimenter’s left hand (which would brush the dummy hand); in this way, the sound source would be as close as possible to the point of contact between brush and dummy hand and would move along with the brush. The sound consisted of a sequence of Matlab-generated white noises at maximum volume, with frequency = 1 Hz and possible durations of 300 ms or 500 ms, which was randomly assigned by Matlab (version 2021b) and visible in advance to the experimenter through the visual cues on the screen. The experimenter delivered longer or shorter brushes accordingly.

#### Questionnaires

In addition to proprioceptive drift measures, a simple 1-item questionnaire was administered after each trial to rate the intensity of the illusory self touch (reported in Table 3). To assess the reliability of each participant’s answers, a final question was asked at the end of the experiment to rate suggestibility (see Table 4. Both questionnaires were readapted from^37^.

**Table 3:** Intensity of illusory self-touch questionnaire. For a child’s answers to the illusory self-touch questionnaire to be considered valid, the suggestibility question had to be rated no higher than 2.

Did you have the impression that — with my help — you were brushing your right hand?	1	2	3	4	5
Rating meaning	not at all	a little bit	yes, to an extent	yes, a lot	yes, absolutely

**Table 4:** Suggestibility question administered at the end of each experiment to assess the reliability of the intensity questionnaire responses.

Did you feel at any point that you had grown a third hand?	1	2	3	4	5
Rating meaning	not at all	only a little bit	yes, sometimes	yes, a lot	yes, absolutely

### Analysis

Proprioceptive precision was obtained from the baseline pointing trials and measured as the logarithm of the inverse if the variance, to normalize extreme values. All continuous variables (age, hand size, and proprioceptive precision) were centered to the grand mean in linear mixed-effects models. Additionally, in all linear mixed-effects models, subjects were included as a random intercept to account for repeated measurements and hand size was added as a covariate.

#### The pointing task

Proprioceptive drift was calculated on a subject-by-subject basis as the difference between the mean of the pointing trials performed after each condition (Fig. 2 b) and the mean of the baseline pointing trials (Fig. 2 a), i.e., the distance between the positions where one’s right index finger was perceived before and after the experimental manipulation (as shown by the red arrow in Fig. 2 c). A shift towards the position of the dummy hand would have a positive value, while a shift in the opposite direction would have a negative value. It should be noted that the final analysis was conducted on over 95% of the trials, as fewer than 5% of the trials were missing. Three SVI participants and two sighted ones performed fewer trials than requested; however, all participants performed at least two out of three expected trials per condition.

The analyses described below were carried out in both the sighted and the SVI group and were performed with RStudio (version 2022.7.1). The SVI group included both blind (n = ten) and low vision children (n = three) as the linear mixed-effects analysis conducted to assess possible differences in proprioceptive drift between them revealed no significant main effect of group, condition or interaction (p’s>.42). First, Shapiro-Wilk normality tests were conducted to test whether baseline pointing values and proprioceptive drift values deviated from normality. Before comparing proprioceptive drift values against zero, we tested for a bias in the average baseline pointing values within each group. In both groups, baseline pointing values were not normally distributed (sighted: W = 0.97, p = 0.01; SVI: W = 0.90, p < 0.01), so an exact Wilcoxon signed rank test was chosen to test for pointing bias. Regarding proprioceptive drift, its values did not deviate from normality in sighted children (tactile SRHI: W = 0.97974, p = 0.6233; audio-tactile SRHI: W = 0.96758, p = 0.2604) or in children with SVI (tactile SRHI: W = 0.949, p = 0.3355; audio-tactile SRHI: W = 0.9338, p = 0.3817). Proprioceptive drift was first examined in each condition to determine whether it differed from zero. For conditions showing non-significant drift, Bayesian one-sample t-tests were conducted to quantify evidence for the null hypothesis, with Bayes Factors 10 interpreted following^53^. After assessing the presence of proprioceptive drift group-wise, we proceeded to compare perceived position of the index finger across conditions (baseline, and after tactile and audio-tactile SRHI, respectively). Prior to fitting mixed-effects models, the relationship between age and perceived hand position was evaluated for linearity using comparisons of linear versus quadratic models and segmented regression to test for potential age-related breakpoints. Subsequently, a linear mixed-effects model was fitted to the raw pointing data to test how condition (baseline, tactile SRHI, audio-tactile SRHI), mean-centered age, and their interaction predict perceived hand position. Subjects were included as a random intercept to account for repeated measurements and hand size was added as a covariate. Type III ANOVA (Satterthwaite’s method) was used to assess the significance of fixed effects, and age slopes for each condition were estimated to facilitate pairwise comparisons between conditions. In the sighted group, proprioceptive drift values were investigated with a linear mixed-effects model as a function of proprioceptive precision, age and their interaction. An initial model including condition and its interactions was compared to a reduced model excluding condition. Nested model comparisons using likelihood ratio tests, along with inspection of AIC and BIC values, indicated that inclusion of condition did not improve model fit. The more parsimonious model without condition was therefore retained as the final model.

For between groups analyses, Levene’s test for equality of variances was employed prior to fitting the linear-mixed effect analysis and revealed no evidence of heteroscedasticity between baseline pointing values of sighted and SVI children (F(1, 55) = 0.41, p = 0.52), suggesting a similar consistency between participants between the two groups. Moreover, Wilcoxon rank sum tests with continuity correction were employed to compare precision and bias (mean value) of the baseline pointing between groups. For all following analyses involving linear mixed-effects models, significance of fixed effects was assessed using Type III ANOVA with Satterthwaite’s method for degrees of freedom. For analyses using standard linear regression, Type III ANOVA was used to evaluate the significance of model terms. A linear mixed-effects analysis was conducted to test the three-way interaction between group, condition and age on raw pointing data while accounting for repeated measures within participant and controlling for hand size. Additionally, proprioceptive precision (mean-centered) was modeled using a linear regression with group, mean-centered age, and their interaction as predictors, and hand size as a covariate. Significance of model terms was assessed with Type III ANOVA. Subsequently, a linear mixed-effects analysis was conducted to examine whether proprioceptive drift was predicted by proprioceptive precision (mean-centered), age (mean-centered), and group, including interactions between group and age and between group and precision to test whether developmental trajectories and precision-related effects differed across groups. As nested model comparisons using likelihood ratio tests, AIC and BIC values showed that condition and its interactions did not significantly improve model fit, it was excluded from the final model for parsimony. Hand size was included as a covariate, and subject was modeled as a random intercept.

#### Questionnaires

For within group analyses, Shapiro-Wilk tests were conducted to test for normality and indicated that the data were not normally distributed in sighted children (tactile SRHI: W = 0.83, p < 0.0001; audio-tactile SRHI: W = 0.81, p < 0.0001) nor SVI children (tactile SRHI: W = 0.78, p < 0.0001; audio-tactile SRHI: W = 0.79, p < 0.0001). Separate Wilcoxon signed-rank tests for tactile and audio-tactile SRHI were then conducted to compare mean responses to the intensity of illusory self-touch questionnaire against a score of 2, which corresponded to the answer ‘a little bit’.

For between group analyses, Wilcoxon rank sum tests were conducted to compare questionnaire responses between sighted and SVI children. Subsequently, linear mixed-effects models were conducted to investigate how trial-by-trial values of proprioceptive drift predicted their respective questionnaire responses depending on the group, with age and hand size as covariates and subject as a random intercept. Finally, linear mixed-effects models with age and hand size as covariates and subject as a random intercept were fit to test how proprioceptive precision predicted first drift values and then questionnaire responses, with group as an interaction term. Here, trial level values of drift were employed to model their association with the respective questionnaire ratings.

## Results

### Experiment 1

#### Pointing task in sighted children

**Fig. 3:**
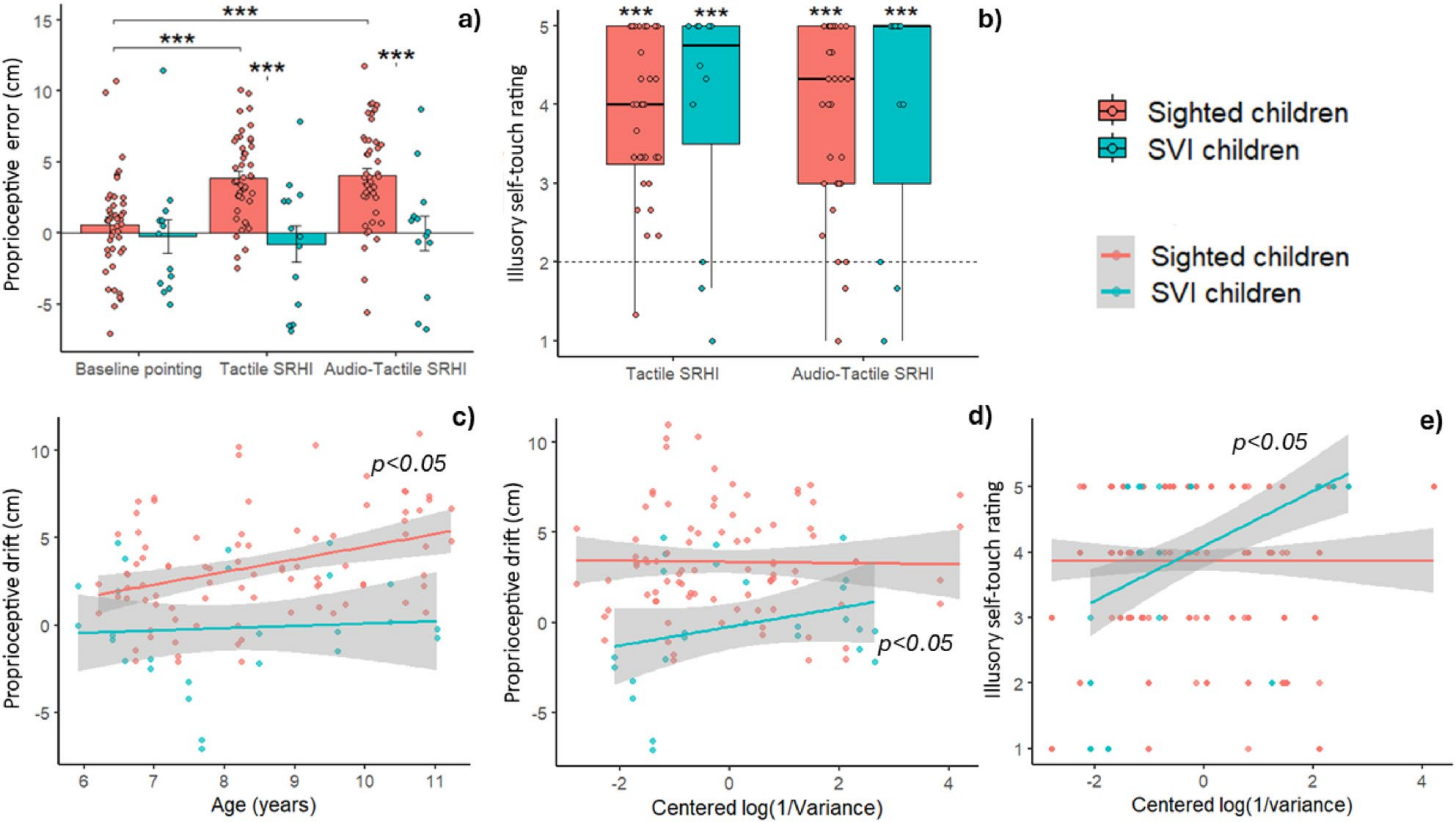
(**a**) Proprioceptive error before and after the Somatic Rubber Hand Illusion (SRHI) in sighted and SVI children. (**b**) Illusory self-touch ratings following the SRHI. (**c**) Effect of age on proprioceptive drift, showing the group X age interaction. Age was centered on the grand mean for use in the interaction model, but is displayed in its original (untransformed) form in the plot for ease of interpretation. (**d**) Effect of proprioceptive precision on drift, showing the group $\times$ precision interaction. (**e**) Effect of precision on intensity of illusory self-touch, showing the group $\times$ age interaction.

The paradigm successfully induced proprioceptive drift: perceived hand position shifted toward the dummy hand in both the tactile (t(43) = –7.59, p < 0.001) and audio-tactile conditions (t(43) = –6.73, p < 0.001, Bonferroni-corrected). The relationship between age and drift was linear; neither quadratic nor segmented models improved fit (all p>.35). Baseline pointing was examined to assess if there was a group-level bias. As baseline deviated from normality, an exact Wilcoxon signed-rank test showed no bias in sighted children (V = 589, p = 0.28). A linear mixed-effects model including condition (baseline, tactile SRHI, audio-tactile SRHI), mean-centered age, their interaction, and hand size as covariate revealed a significant main effect of condition (F(2,75.2) = 40.39, p < 1e-12) and a condition $\times$ age interaction (F(2,75.2) = 4.35, p = 0.016), but no main effects of age (F(1,37) = 1.25, p = 0.27) or hand size (F(1,36.95) = 0.23, p = 0.636). Pairwise comparisons indicated that the age-related slope in the tactile condition versus baseline approached significance (estimate = –0.60, SE = 0.28, p = 0.084), and the audio-tactile slope differed significantly from baseline (estimate = –0.78, SE = 0.28, p = 0.017); however, tactile and audio-tactile slopes did not differ from each other (estimate = –0.18, SE = 0.28, p = 0.79). These results show that SRHI induces a bias in perceived hand position (i.e., proprioceptive error) in sighted children (figure 3 a). To explore predictors of proprioceptive drift (perceived position of the hand following SRHI – baseline pointing position), nested linear mixed-effects models compared age, precision (mean-centered log(1/variance) of baseline pointing), and condition (tactile and audio-tactile). Including condition did not improve fit (AIC = 376–381, BIC = 393–404; likelihood ratio tests all p > 0.37), so it was excluded. In the final model, age significantly predicted drift (F(1,40.1) = 4.25, p = 0.046), while precision (F(1,40.3) = 1.25, p = 0.27) and the age $\times$ precision interaction (F(1,40.1) = 0.50, p = 0.48) were not significant.

#### Questionnaires in sighted children

To assess the reliability of participants’ answers depending on individual suggestibility, at the end of the experimental session participants were made to rate an impossible statement. Only participants whose suggestibility score was smaller than two were included in these analyses. Wilcoxon signed-rank tests revealed that responses to the reported intensity of illusory self-touch were significantly higher than the value of two (corresponding to: ‘a little bit’) in sighted children (tactile SRHI: V = 4691, p < 0.001; audio-tactile SRHI: V = 4308.5, p < 0.001). No differences between conditions were observed (W = 532, p = 0.73). Of the 36 children included in the analysis, the children whose average rating was below two were one in the tactile condition and two in the audio-tactile one.

### Experiment 2

#### Pointing task in SVI children

First, we assessed whether the experimental conditions had determined a shift in perceived hand position in SVI children. T-tests revealed that proprioceptive drifts following tactile SRHI and audio-tactile SRHI were not significantly different from 0 (p’s> 0.51) and no difference between the two conditions was found (p = 0.21). Considering the absence of evidence supporting H1, we assessed the evidence in favour of H0 given the present data. We performed Bayesian one-sample t-tests using a default Cauchy prior with a scale of 0.707^53^ to compare proprioceptive drift (perceived position of the hand following SRHI – baseline pointing position) with 0, where Bayes Factors 10 > 1 indicate more evidence in favor of the alternative hypothesis and Bayes Factors 10 < 1 provide support for the null one (to consult the reference table, see^54^). For the tactile SRHI condition, the present data was 3 times more likely under the null hypothesis ($\upmu$ = 0), with a Bayes Factor of 0.34 (BF10 = 0.34, r = 0.707). Similarly, for the audio-tactile SRHI we found moderate evidence in favor of the null hypothesis, with a Bayes Factor of 0.28 (BF10 = 0.28, r = 0.707); also, when the two conditions were compared, anecdotal evidence was found in support of the null hypothesis (BF10 = 0.41, r = 0.707). As was the case for sighted children, baseline pointing values deviated from normality, so an exact Wilcoxon signed rank test was used to test the pointing error and showed not significant difference from 0 (V = 34, p = 0.44), suggesting no systematic bias in this group. Linearity of the relationship between age and perceived hand position was assessed separately for each experimental condition. Linear versus quadratic models were compared; adding a quadratic term did not improve model fit in either condition (all ps <.24) and segmented regression models did not significantly improve fit over linear models (ps <.22), showing no evidence for curvature or slope changes. We then proceeded to fit a linear mixed-effects model to test how condition (including baseline, tactile SRHI and audio-tactile SRHI), age (mean-centered) and their interaction predict perceived hand position, adding hand-size (mean-centered) as a covariate and subject as a random intercept. Type III ANOVA indicated no significant main effects of condition (F(2, 16) = 0.94, p = 0.41) or age (F(1, 7) = 0.11, p = 0.75), and no significant condition $\times$ age interaction (F(2, 16) = 0.86, p = 0.44). The main effect of hand size was also non-significant (F(1, 7) = 0.34, p = 0.58). These results show no evidence that either the tactile or the audio-tactile SRHI paradigms induced any change in where SVI children perceive their hand, which was, on average, its correct location, as no systematic bias was found (p = 0.44).

#### Questionnaires in SVI children

As one child rated highly an impossible statement, 12 children were included in the analysis. Wilcoxon signed-rank tests revealed that responses to the reported intensity of illusory self-touch were significantly higher than the value of 2 (tactile SRHI: V = 432, p = < 0.001; audio-tactile SRHI: V = 386, < 0.001), a finding which corresponded with observable surprise in children upon explanation of the paradigm, suggesting that the reported perceptual effects were meaningful. However, three children (25%) rated strength of illusory self touch below 2 in both conditions.

#### Pointing task - comparison between groups

We fit a linear mixed-effects model predicting perceived hand position (i.e., proprioceptive error) with group (sighted vs. SVI), condition (baseline pointing, tactile SRHI, audio-tactile SRHI), and age (mean-centered) as fixed effects, including all interactions, hand-size as a covariate and a random intercept for subject. Type III ANOVA (Satterthwaite’s method) indicated significant main effects of group (F(1, 44.99) = 7.78, p = 0.0077) and condition (F(2, 91.10) = 3.87, p = 0.024), as well as a significant group $\times$ condition interaction (F(2, 91.10) = 13.52, p = $7.2 \times 10^{-6}$). Neither age (F(1, 44.99) = 0.54, p = 0.47) nor the group $\times$ age (F(1, 44.99) = 0.15, p = 0.70), condition $\times$ age (F(2, 91.09) = 1.46, p = 0.24), or group $\times$ condition $\times$ age interactions (F(2, 91.09) = 1.83, p = 0.17) were significant. Pairwise comparisons were carried out to investigate the significant interaction and showed that sighted children perceived their hand to be significantly closer to the dummy hand compared to SVI children following both tactile and audio-tactile SRHI (tactile: $\Delta$ = 5.18, SE = 1.46, t = 3.56, p = 0.0007; audio-tactile: $\Delta$ = 5.26, SE = 1.46, t = 3.61, p = 0.0006), whereas no localization error was found in the baseline condition ($\Delta$ = 0.77, SE = 1.46, t = 0.53, p = 0.60). Comparisons between conditions showed a significant difference from baseline in sighted children (baseline pointing vs. tactile SRHI: $\Delta$ = −3.244, SE = 0.434, t(91) = −7.482, p < 0.0001; baseline pointing vs. audio-tactile SRHI : $\Delta$ = −3.563, SE = 0.438, t(91) = −8.138, p < 0.0001), with no difference between tactile and audio-tactile conditions ($\Delta$ = −0.319, SE = 0.438, t(91) = −0.730, p = 0.7466). No significant difference was found in the SVI group (all ps >.38). The effect of hand size was non-significant (F(1, 44.97) = 0.48, p = 0.49).

We then tested whether proprioceptive precision (mean-centered) was predicted by age (mean-centered), group, and their interaction, adding hand size as a covariate. Proprioceptive precision resulted to increase with age across the sample ($\beta$ = 0.677, SE = 0.307, F(1,45) = 4.87, p = 0.033), indicating that older children were generally more precise. There was no significant difference in precision between sighted and SVI children (F(1,45) = 0.19, p = 0.66), and the age $\times$ group interaction did not reach significance ((F(1,45) = 2.08, p = 0.156). Hand size was also not a significant predictor (F(1,45) = 0.16, p = 0.69). We then proceeded to examine the predictors of individual proprioceptive drift across groups. Model comparison showed that adding condition (tactile, audio-tactile) —either as a covariate or in interaction with group, age, and precision —did not improve model fit. The condition-as-covariate model did not differ significantly from the reduced model ($\Delta$AIC = 0.77; $\Delta$BIC = 3.37; $\chi _{2}$(1) = 1.23, p = 0.27), and the full model with interactions similarly failed to improve fit ($\Delta$AIC = 6.92; $\Delta$BIC = 22.50; $\chi _{2}$(5) = 3.84, p = 0.57). Proprioceptive drift was analyzed with a mixed-effects model (random intercepts for subjects). The analysis confirmed a significant effect of group already observed, with sighted children showing higher drift than SVI children (F(1,50) = 20.21, p < 0.001), and a significant interaction of age $\times$ group (estimate = –1.43, SE = 0.69, p = 0.043), with a significantly steeper slope in the sighted group. Additionally, there was a marginal effect of precision on drift (F(1,50) = 2.91, p = 0.094) and significant interactions of precision $\times$ group (estimate = 1.55, SE = 0.64, p = 0.019). Post-hoc comparisons showed that only in SVI children precision significantly predicted proprioceptive drift (slope = 1.28, 95% CI [0.14, 2.42]), but not in sighted children (slope = −0.27, 95% CI [−0.85, 0.31]). This group difference was confirmed by a significant slope contrast (SVI vs sighted: estimate = 1.55, SE = 0.64, t = 2.42, p = 0.019), consistent with the interaction effect. Hand size did not significantly affect drift (F(1, 49.93) = 1.48, p =.23). To confirm that the primary findings were not driven by influential observations or distributional violations, the model was re-estimated using robust linear mixed-effects models^55^, including the seven previously excluded sighted participants. Because robust models do not yield p-values, effects were evaluated based on the magnitude of the robust t-statistics, with |t| $\ge$ 2 treated as indicating meaningful support. In addition, 95% Wald confidence intervals were computed from the robust standard errors ($\beta$ ± 1.96 $\times$ SE). The group difference in proprioceptive drift remained evident ($\beta$ = 4.52, 95% CI [2.19, 6.84], robust t = 3.81). The association between proprioceptive precision and drift in the SVI group was preserved ($\beta$ = 1.34, 95% CI [0.11, 2.58], robust t = 2.13), and the precision $\times$ group interaction remained supported ($\beta$ = –1.76, 95% CI [–3.13, –0.40], robust t = –2.53), indicating differential precision–drift associations across groups, consistent with the primary analysis. The age $\times$ group interaction was attenuated ($\beta$ = 1.42, 95% CI [–0.06, 2.90], robust t = 1.88) but trended in the expected direction.

#### Questionnaires - comparison between groups

Wilcoxon rank sum tests were conducted to compare questionnaire responses between sighted and SVI children, yielding no statistically significant differences (p’s >.7). Linear mixed-effects analyses showed that trial-by-trial drift was a marginal predictor of questionnaire ratings ($\beta$ = 0.072, SE = 0.038, p =.062), and the drift $\times$ group interaction also reached marginal significance ($\beta$ = –0.075, SE = 0.041, p =.072). The estimated drift–response slope was positive in SVI children ($\beta$ = 0.072), whereas sighted children showed no measurable drift effect ($\beta$ = –0.003). The difference between slopes approached significance ($\beta$ = 0.075, SE = 0.042, t(213) = 1.80, p = 0.073). Proprioceptive precision significantly predicted questionnaire ratings ($\beta$ = 0.504, SE = 0.208, p =.021), with a significant precision $\times$ group interaction ($\beta$ = –0.582, SE = 0.246, p =.023): post-hoc comparisons showed that the precision-rating association was significant only in SVI children (slope = 0.50, 95% CI [0.08, 0.93]), but not in sighted children (slope = −0.08, 95% CI [−0.35, 0.20]). The difference in slopes between groups was significant (SVI vs sighted: estimate = 0.58, SE = 0.25, t = 2.37, p = 0.023), consistent with the interaction effect observed. Finally, we re-estimated the questionnaire model using robust mixed-effects regression, including the seven sighted participants previously excluded as outliers. We examined whether trial level values of proprioceptive drift and proprioceptive precision, and their interactions with group, predicted questionnaire ratings. As in the drift model, 95% Wald confidence intervals were computed from the robust standard errors ($\beta$ ± 1.96 $\times$ SE). The precision effect in the SVI group remained supported ($\beta$ = 0.50, 95% CI [0.05, 0.95], robust t = 2.17), as did the precision $\times$ group interaction ($\beta$ = –0.60, 95% CI [–1.11, –0.10], robust t = –2.33), confirming that the association between precision and questionnaire ratings differed between groups. Consistent with the primary analysis, the main effect of proprioceptive drift was weak ($\beta$ = 0.055, 95% CI [–0.01, 0.12], robust t = 1.71), whereas the drift $\times$ group interaction (marginally significant in the main analysis) was meaningfully supported ($\beta$ = –0.069, 95% CI [–0.14, –0.003], robust t = –2.05). Main effects of age, hand size, and group were small and not meaningfully supported (|t| < 1). Overall, the key moderation effects were preserved under robust estimation, and the corresponding confidence intervals largely excluded zero for the interaction terms, indicating that the observed moderation patterns were unlikely to be driven by influential observations.

## Discussion

In the present study, we administered the Somatic Rubber Hand Illusion (SRHI) to sighted and Severally Visually Impaired (SVI) children (6–11 years old). In experiment 1, sighted children reported significant illusory self-touch and their perceived hand position shifted towards the dummy hand (proprioceptive drift) revealing a flexible representation of their hand position. In sighted children, proprioceptive drift towards the dummy hand was revealed to increase with age, replicating a previous result on the tactile SRHI in a similar age-group^37^, a result which stands in contrast to what is typically shown to be the developmental pattern of proprioceptive drift in the visual rubber hand illusion^35,38^. Contrary to our hypothesis, no incremental effect of sound was found regardless of age, which would suggest that, unlike adults^30^, children within this age range do not utilize auditory information when computing the position of their unseen hand. While children already perform optimal visuo-haptic integration at the age of eight^43^, the present result would suggest a later developing mechanism for auditory modulation on own hand localization, in accordance with the finding that optimal audio–proprioceptive integration for size judgments develops only in early adolescence in sighted individuals^11^. Experiment 2 showed that, in accordance with our hypothesis, proprioceptive drift was significantly different between groups, regardless of the presence of auditory cues, as SVI children did not show proprioceptive drift, with Bayesian statistics showing moderate support in favor of the null hypothesis in both conditions. It should be noted that our implementation of the experimental paradigm was designed to maximize the potential to detect proprioceptive drift in the SVI group, despite the expectation of a negative result. Drawing from paradigms known to enhance proprioceptive drift in sighted adults, we incorporated features aimed at delivering highly effective stimulation, i.e., variable spatiotemporal patterns of tactile stimulation and synchronous auditory cues^29,30^. In light of these considerations and of the bayesian support for a null result, we conclude that the SRHI paradigm fails to introduce a bias in pointing-based hand localizations in SVI children. While the significant age $\times$ group interaction suggests different developmental patterns between groups, with a steeper increase in proprioceptive drift in sighted children, given the smaller SVI sample, this group-specific pattern should be considered exploratory.

Surprisingly, and contrary to prior hypotheses, questionnaire results showed that both sighted and SVI children experience illusory self-touch, showing that the majority of SVI children believed that they were brushing their own hand instead of the dummy. This result is, to the best of our knowledge, the first report of illusory self-touch being induced by SRHI in an SVI population when naïve to the set-up. At first glance, this surprising result would seem further evidence that proprioceptive drift and illusory self-touch are relatively segregated processes^56–58^. In opposition with this, we found a positive association between proprioceptive drift and illusory self-touch ratings in SVI children. This suggests a generally more liberal response style in the latter measure, rather than a lack of correspondence with proprioceptive drift. On a group level, SVI children display no proprioceptive drift towards the dummy hand, which extends to children the results found in blind adults^5,20,45^. Our first hypothesis to explain this, based on findings of enhanced precision in the spared senses^2,46^, revolved around the idea that blindness would favor the development of a heightened proprioceptive precision, making the spatial discrepancy between the real hand and the dummy one too obvious to ignore when performing own hand localization. Had the SVI group displayed a striking superiority in proprioceptive precision in the baseline pointings (prior to illusion induction), their lack of drift may be interpreted as a sign of higher reliance on their proprioceptive signals^59^. In the present sample, however, proprioceptive precision is not significantly different between groups, as was also shown in blind and sighted adults^20^: this makes it an inadequate candidate to explain, on its own, this group difference. In sighted children, higher proprioceptive precision did not reduce susceptibility to cross-modal modulation —neither in proprioceptive drift nor in illusory self-touch— indicating that, in typical development, proprioceptive recalibration becomes robust to individual differences in proprioceptive precision. This highlights that the relationship between proprioceptive precision and drift is more complex than a straightforward Maximum Likelihood Estimate (MLE)-based account would suggest^60^. In accordance with this, previous work in sighted adults showed that in the RHI, ownership ratings were not significantly linearly associated with precision^42^ and their relationship with proprioceptive uncertainty was not fully captured by the MLE model^41^. In contrast, in SVI children, proprioceptive precision predicted both proprioceptive drift and illusory self-touch, but in the opposite direction from our initial hypotheses: higher precision was associated with greater drift toward the dummy hand and stronger illusory self-touch ratings. It would seem, therefore, that while proprioceptive drift is robust in sighted children across levels of proprioceptive precision, a minimal level of proprioceptive reliability may be necessary for effective recalibration in the SVI group. Although the group-level drift was near zero, individual variability indicates that some SVI children recalibrated hand position toward the dummy hand, while others drifted further away. These findings imply that an additional factor modulates the relationship between precision and recalibration in SVI children. We suggest that effective recalibration requires detecting the spatial discrepancy between the two hands, which depends on transforming proprioceptive information into external spatial coordinates. Because the pointing task requires estimating hand location in external space, higher precision in this task suggests higher spatial mapping abilities, which may drive proprioceptive drift as a mechanism to resolve the sensory conflict induced during the SRHI^57,61^. It was first suggested by Petkova et al.^20^ that the explanation for the absence of drift in blind adults might lie in their different representation of external space^62–65^. Accordingly, blind individuals have been shown to perform spatial tasks equally well when their hands are crossed over the body midline, a posture that reduces performances of sighted participants; this lack of a so-called crossed-hands deficit reveals a different interplay between body position and external space, which has been attributed to a difficulty in remapping body-centered coordinates onto external space^6,20,66,67^. Consistently, SVI children may have difficulty remapping their body position onto an external reference frame^4^, which could translate into their failure to show proprioceptive drift. Our results suggest that, in this population, proprioceptive precision in hand localization may act as a gate on the development of the spatial competence required for proprioceptive drift, thereby shaping cross-modal calibration on proprioception itself. In this perspective, the lack of proprioceptive drift that we observed in SVI children may reflect differences in the ability to remap perceived hand position of one’s own hand onto a location in external space which would optimally reduce the presented sensory conflict. In conclusion, SVI children show preserved illusory self-touch experiences during SRHI, but these experiences do not translate into systematic external-space localization drift in this paradigm. On a behavioral level, the present findings represent a first window into how visual experience shapes perception of own body in space and suggest that proprioceptive training may provide a promising approach to enhance spatial awareness.

### Limitations

Due to time constraints in testing children with severe visual impairment (SVI), and in order to maintain procedural consistency across groups, we did not include an asynchronous stimulation condition. Consequently, we cannot conclusively attribute the observed effects to multisensory synchrony per se. Furthermore, we did not assess children’s ability to localize sounds in external space, which limits our ability to determine a possible role of variability in spatial hearing rather than multisensory body representations. At the same time, caution is warranted in interpreting the illusory self-touch findings. The questionnaire measure we adopted (adapted from^37^) may have inflated reports of illusory self-touch, as the midpoint of the scale was not neutral. Despite the use of a control suggestibility question and the removal of suggestible participants, such asymmetric labeling could bias responses toward agreement, potentially amplifying illusion reports. Therefore, the findings about illusory self-touch should be interpreted carefully.

## Data Availability

The datasets generated and analysed during the current study are available in the MYSpace – H2020 ERC repository on Zenodo, https://doi.org/10.5281/zenodo.18019773. Data access inquiries may be directed to Lara Coelho at lara.coelho@iit.it.
